# Supplementary material for: A cryo-electron tomography study of ciliary rootlet organization
Source: eLife. 2024 Dec 6;12:RP91642. doi: 10.7554/eLife.91642 (PMC11623930; doi:10.7554/eLife.91642)
Supplement: Supplementary file 1. — (a) Particle distribution of subTOM classification for particle heterogeneity. (b) Particle distribution of RELION 4.0 Alpha classification with alignment. [file elife-91642-supp1.docx]

## Supplementary File 1—Tables

Supplementary File 1a. Particle distribution of subTOM classification for particle heterogeneity.

| class | number of particles | percentage of total |
| --- | --- | --- |
| 1 | 55,655 | 9.41% |
| 2 | 71,118 | 12.02% |
| 3 | 85,899 | 14.52% |
| 4 | 20,524 | 3.47% |
| 5 | 23,219 | 3.93% |
| 6 | 79,199 | 13.39% |
| 7 | 55,608 | 9.40% |
| 8 | 30,798 | 5.21% |
| 9 | 33,461 | 5.66% |
| 10 | 135,972 | 22.99% |
| All | 591,453 | 100.00% |

Supplementary File 1b. Particle distribution of RELION 4.0 Alpha classification with alignment.

| class | number of particles | percentage of total |
| --- | --- | --- |
| 1 | 49,934 | 27.71% |
| 2 | 32,221 | 17.88% |
| 3 | 28,871 | 16.01% |
| 4 | 34,736 | 19.13% |
| 5 | 34,490 | 19.27% |
| All | 180,252 | 100.00% |
